# Influence of the Sense of Coherence, the Doctor–Patient Relationship, Optimism and Non-Haematological Adverse Reactions on Health-Related Quality of Life in Patients with Breast Cancer

**DOI:** 10.3390/jcm8122043

**Published:** 2019-11-21

**Authors:** Blanca Prieto-Callejero, Francisco Rivera, Montserrat Andrés-Villas, Juan Gómez-Salgado

**Affiliations:** 1 Hospital Virgen de la Bella, 21440 Lepe, Spain; blanca.prietoc@gmail.com; 2 Department of Experimental Psychology, Faculty of Psychology, University of Seville, 41018 Seville, Spain; 3 Department of Social, Developmental and Educational Psychology, Faculty of Education Sciences, University of Huelva, 21071 Huelva, Spain; montserrat.andres@dpsi.uhu.es; 4 Department of Sociology, Social Work and Public Health, University School of Social Work, University of Huelva, 21071 Huelva, Spain; jgsalgad@gmail.com; 5 Safety and Health Postgraduate Programme, Universidad Espíritu Santo, Guayaquil 091650, Ecuador

**Keywords:** health-related quality of life, breast cancer, sense of coherence, optimism, toxicities

## Abstract

Breast cancer is the most common malignant tumour in women around the world. The objective of this study was to quantify the number of non-haematological adverse reactions associated with chemotherapy, as well as to assess the effect of the sense of coherence, optimism–pessimism and the quality of the doctor–patient relationship on the quality of life of breast cancer patients. To this end, a cross-sectional descriptive study was conducted involving 110 breast cancer patients who were treated with docetaxel, epirubicin, and cyclophosphamide during the period 2012–2014. The difference in the quality of life in patients who have five or fewer toxicities compared to those with more than six is highlighted. This difference is not as important when comparing patients with 6 to 10 toxicities and those with more than 10. The multivariate model used in this study corroborates the direct implication of the sense of coherence on the quality of life and adds the number of adverse reactions as a new construct. This has virtually the same impact on the quality of life of these patients, but in reverse. In conclusion, to improve the quality of life of breast cancer patients it would be necessary to have an impact on the number of adverse reactions involved in chemotherapeutic treatment, as well as on psychological interventions, with the sense of coherence as a possible starting point.

## 1. Introduction

Breast cancer is the most common malignant tumor in women worldwide. In Spain, more than 20,000 cases are diagnosed per year (26,370 in 2017). By 2019, the incidence of breast cancer in Spain is estimated at 32,536 new cases [1].

Women with breast cancer have an increased risk of suffering psychological problems, such as depression and distress, compared to healthy control cases. Often, these psychological problems remain untreated in the usual clinical practice [2,3,4]. In this sense, there is a high demand for psychosocial care for these types of patients [5].

The early detection of breast cancer [6], and interventions to promote psychological adjustment along the cancer pathway, should be the main focus in breast cancer interventions [4].

There are several aspects to consider in these types of patients, which could be very useful in easing the way through their process, such as the sense of coherence, the doctor–patient relationship, optimism, and the presence of adverse events, particularly non-haematological ones. Therefore, in the present study, the assumption was made that the perceived sense of coherence, pessimism or optimism, and the quality of the patient–doctor relationship influence breast cancer patients’ quality of life and adverse effects.

In this sense, considering the doctor–patient relationship perspective, dialogue is considered to be an interactive communication to which a curative, educational, and preventive role is attributed [7]. However, training in this area is intuitive and unstandardised, although generally acquired from the experience of professional practice [8].

In addition, it should be considered that what patients know about their disease is not the exclusive result of what information the doctor gives them. Therefore, informing the patient does not guarantee that the patient has understood the provided information. In this way, it can be concluded that the relationship with the patient and her collaboration during treatment could depend on the manner of reporting [4,9].

Thus, a good doctor–patient relationship is important as it determines the scope of the information that patients will give to the doctor, the patients’ satisfaction, their compliance with the treatment, and even their results [4,10].

Regarding the sense of coherence, it is a particularly relevant factor for the development and maintenance of human health and it seems to be a factor for strengthening resilience and a good basis for the development of health promotion actions [11,12].

The sense of coherence can therefore be a protective factor against demoralisation in the context of a serious disease such as cancer [13]. It is therefore a useful tool for identifying people who are particularly vulnerable to distress, anxiety, and depression [14].

In addition, regarding the coping strategies employed by breast cancer patients during their treatment and recovery process, patients with higher optimism have proven to be more likely to use coping strategies [15,16,17]. Optimism is one of the most relevant psychological variables associated with good quality of life in breast cancer patients [18]. There are studies [19] that support the existence of a close relationship between optimism and the immune system, with optimism strengthening the system against stressful situations. However, pessimism is associated with the opposite effect, and is also related to disruption of social activities and enjoyment, and to difficulties in adapting to the diagnosis and treatment, thus increasing the risk of adverse psychological reactions [18].

As for chemotherapeutic treatments, they have evolved significantly [20]. Several clinical trials have proved the efficacy of taxane treatment in breast cancer patients [21,22,23]. However, these treatments involve significant haematological and non-haematological toxicity.

With regard to the assessment of non-haematological adverse reactions related to chemotherapy, this is subjective as it depends on the depth of questioning by the oncologist and the information that the patient spontaneously provides during the consultation. However, Palacios-Espinosa [4] has highlighted how adverse reactions are associated with a lack of adherence to this type of cancer treatment. This study attempts to assess the number of adverse reactions associated with chemotherapy experienced by breast cancer patients during their treatment.

However, despite its relevance, few studies [24,25,26] have shown the impact of adverse reactions and psychological aspects on the quality of life of patients with breast cancer. Most studies deal with nausea, vomiting, lymphedema, and pain. However, they do not consider other toxicities, such as peripheral neuropathy [25], which are equally relevant for the patient.

Likewise, in Spain, as the figures for women who suffer this type of cancer are gradually increasing, a study on health-related quality of life should imply a highly relevant line of research. A review, conducted in our country [24], on breast cancer patients’ quality of life revealed the lack of research on this subject, despite generalised recommendations to include this parameter as an outcome in clinical studies. However, there is abundant international information on this field of study that, although referring to the same type of patient, could not be applied in our country as it relates to a concept that deals with the perceptions associated with the immediate and specific reality of each woman.

Considering this, the objective of this study was to quantify the number of non-haematological adverse reactions associated with chemotherapy as well as to assess the effect that the sense of coherence, optimism–pessimism, and quality of the doctor–patient relationship has on the relationship between the quality of life and the adverse symptomatology of the patient.

## 2. Materials and Methods

### 2.1. Design

A cross-sectional descriptive study was conducted in a second-level hospital on all breast cancer patients receiving first-line neoadjuvant or adjuvant treatment with docetaxel, epirubicin and cyclophosphamide. The total study period was from May 2012 to August 2014.

### 2.2. Participants

Breast cancer patients on chemotherapeutic first-line neoadjuvant or adjuvant treatment with the TEC regimen (docetaxel 75 mg/m^2^ + epirubicin 75 mg/m^2^ + cyclophosphamide 500 mg/m^2^ + colony stimulating factor (filgrastim or pegfilgrastim) + triple antiemetic therapy with dexamethasone, aprepitant and ondansetron) who had, at least, undergone the first cycle of this regimen were included in the study, with the aim of gathering information on the adverse reaction suffered during the 21 days between the various chemotherapy cycles. Patients who were receiving their first treatment cycle were excluded, despite meeting the inclusion criteria, as they were unlikely to present any adverse reactions at this stage.

For the calculation of the required sample size, the total number of patients with these characteristics and undertaking treatment for one year with the TEC regimen (approximately 60) was considered, giving an estimated representative sample size of 52 patients, with a 95% confidence level and a maximum estimate error of 5%. The final sample included 110 patients.

### 2.3. Instruments

An “ad hoc” questionnaire for the patients was developed, including the possible non-haematological adverse reactions that are frequent in the TEC regimen. These were obtained from Miguel Martin’s publications [21,27]. This was carried out using an expert consultation to determine the completeness and uniqueness of the included indicators. (See Supplementary File 1. Questionnaire.) Likewise, in order to verify its validity, the questionnaire was subjected to previous assessment in a study conducted on the same type of patients.

For assessing the quality of life in relation to health, the EuroQol-5D questionnaire [28] was used as it shows an A level of recommendation and because it best suited the needs of the study. The sense of coherence data were collected through the SOC-13 questionnaire. Several studies have shown the adequate psychometric properties of this scale, both in English and Spanish versions [29,30,31]. In addition, optimism and pessimism were measured though the “Life Orientation Test-Revised” (LOT-R) test for having shown interesting potential for research applied to these psychological constructs [32], as well as adequate reliability and validity levels shown in validation and adaptation studies [24,25,26,27,28,29,30,31,32,33]. There, these psychological constructs are positively related to positive affect and negatively related to neuroticism, perceived stress, and negative affect [32]. As for its reliability, Martínez–Correa et al. [34] show a Cronbach Alpha value of 0.75 in their study.

### 2.4. Procedure

The questionnaire was given at random during one of the six treatment cycles, so that when stratified homogeneously by therapy cycles, a minimum sample of 10/11 subjects was estimated in each of the cycles. In no case did the doctor know which patients were involved in the study or during which cycle they would be surveyed, therefore, the oncologist was always the same doctor (specialist in breast cancer) and acted at all times according to his/her usual clinical practice. The patients had to pre-enter the oncology consultation and then move on to the day hospital unit and receive their treatment. Then, in the day hospital unit, each patient was given the questionnaire that was the subject of study, and she had to provide answers about the previous 21 days, that is, the previous cycle.

The study was carried out following the “Ethical Principles for Medical Research Involving Human Subjects”, compiled in the latest version of the Helsinki Declaration (Edinburgh version, October 2000), for the development and follow-up of this clinical research. In all cases, the anonymity of the participants was guaranteed, having the approval of the Huelva Biomedical Research Ethics Committee for the research protocol. Likewise, the data obtained during the study were processed in accordance with Law 5/1999 and the applicable regulations. An informed written consent was requested prior to participation.

### 2.5. Data Analysis

Descriptive statistics, contrast tests of proportions (Chi-square test), and means (Student *t*-test and Mann–Whitney U test) were used, as well as contrast at the agreement level (Cohen’s Kappa) and effect size (Phi, Cramer’s V, and Cohen’s d).

At the multivariate level, a linear regression analysis was used, based on a stepwise construction model and the structural equation model, following the Maximum Likelihood (ML) estimation method.

The applied statistical software included the IBM SPSS Statistics 22.0 (IBM, NY, USA) and the EQS 6.2. In all cases, a statistical significance of 5% (*p* < 0.05) was required.

## 3. Results

The sample was made up of 110 women. The participant profile for this study was a woman with an average age of 49.61 years (standard deviation: 8.28 years and with an age range from 29 to 68 years), predominantly rural (64.5%), married (78.9%), with children (83.5%), and who is usually accompanied during the consultation (96.2%).

### 3.1. Evaluation of the Number of Toxicities Associated with the TEC Regimen and its Impact on Health-Related Quality of Life

As for the number of toxicities per patient, they are divided into three subgroups: the first would encompass patients with five or fewer toxicities per cycle; the second one, patients with 6 to 10 toxicities; and, finally, the third subgroup would include patients with more than 10 toxicities per chemotherapy cycle.

First, the subgroups were related as a whole to the quality of life, finding a significant difference (K-W (2.72) × 13.334, *p* = 0.001). Later, the Bonferroni correction was applied, followed by Mann–Whitney’s U as a post-hoc test to highlight the difference between the various subgroups. Data are listed in Table 1.

The results show a statistical difference between the first subgroup of patients and the second one (U (56) × 59.5, *p* = 0.001), and between the first and the third subgroups (U (25) = 24.5, *p* = 0.006). Both agree to present a large effect size (Cohen’s d 1.38 and 1.41, respectively). This statistical difference is not found between the second and the third subgroups (U (63) = 287.5, *p* = 0.156), although a small effect size is reported (Cohen’s D = 0.46).

In particular, a higher quality of life is observed in patients with five or fewer toxicities (mean = 16.22), as compared to those with between 6 and 10 toxicities (mean = 14.18) and those with more than 10 (mean = 13.56).

### 3.2. Evaluation of the Number of Toxicities Associated with the TEC Regimen and Its Relationship with the Sense of Coherence and the Dispositional Optimism–Pessimism

On the other hand, the sense of coherence is related to the number of toxicities per patient (Table 2). Globally, there are no statistically significant differences (K-W (2.74) = 4.393, *p* = 0.111). When looking at the relationship between the different subgroups, no statistical differences are observed between them, but both the relationship between the first subgroup and the second one (U (53) = 129, *p* = 0.050) and that between the first subgroup and the third one (U (31) = 62, *p* = 0.72) have a moderate effect size (Cohen’s d = 0.70 and Cohen’s d = 0.77, respectively).

Based on the results of the effect size, it can be determined that there is a tendency to present a greater sense of coherence in those patients with five or fewer toxicities per cycle (mean = 69.80), as compared to those patients with 6 to 10 toxicities (mean = 61.37) or more than 10 toxicities (average = 60.43).

The relationship between optimism and the number of toxicities per patient was then analysed without finding a statistical relationship when assessed as a whole (K-W (2.77) = 2.114, *p* = 0.348). Similarly, this statistical difference between the different subgroups is not found. However, there is a moderate effect size (Cohen’s d = 0.61) between the first subgroup (with five or fewer toxicities) and the third one (with more than 10 toxicities) (U (34) = 91, *p* = 0.287). Specifically, those patients in the first subgroup are more likely to be optimistic (mean = 13.70) than those in the third one.

On the other hand, by analysing the relationship between pessimism and the number of toxicities per patient, statistically significant differences are found (K-W (2.77) = 8.561, *p* = 0.014) when assessed as a whole.

In particular, a statistical difference is found between the second and the third subgroups (U (67) = 304, *p* = 0.005), obtaining a moderate effect size (Cohen’s d = 0.75). Therefore, patients with more than 10 toxicities per cycle show higher pessimism levels (mean = 10.33), as compared to those with between 6 and 10 toxicities (mean = 8.07).

Likewise, despite no statistical differences being observed between the first and the third subgroups (U (34) = 67, *p* = 0.046), a large effect size can be seen (Cohen’s d = 0.82). Specifically, patients in the third subgroup show greater pessimism levels (mean = 10.33) vs. those with five or fewer toxicities per chemotherapy cycle (mean = 7.80).

### 3.3. Assessment of the Number of Toxicities Associated with the TEC Regimen and Its Relationship with the Quality of the Doctor–patient Relationship

Globally, significant differences are found (K-W (2.78) = 8.670, *p* = 0.013). When analysed by subgroups, statistical differences are found between the first subgroup (five or fewer toxicities per patient) and the second one (between 6 and 10 toxicities) (U (54) = 98, *p* = 0.005), and between the first and the third subgroups (more than 10 toxicities) (U (34) = 499, *p* = 0.006). In both cases, a moderate effect size is found. In particular, a greater doctor–patient relationship is observed in those patients in the first subgroup (mean = 44.60) vs. patients belonging to the second subgroup (mean = 41.32) or the third one (mean = 41.63).

### 3.4. Structural Equation Model of the Influence of Adverse Symptomatology and Sense of Coherence on the Patients’ Quality of Life

**Figure 1:**
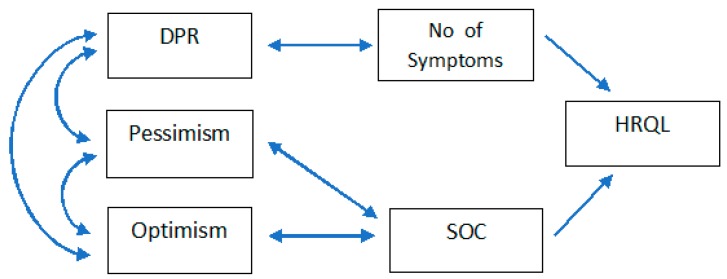
Initial model of the relationship between SOC, the number of symptoms, and HRQL analysed by contrast. DRP: Doctor–patient relationship; SOC: Sense of coherence; HRQL: Health-related quality of life.

Finally, a contrast was made between a multivariate model that proves the joint effect of adverse reactions and the sense of coherence on the quality of life. In turn, the relationship between optimism and pessimism and with a sense of coherence was analysed, as well as the doctor–patient relationship and its influence on the number of adverse symptoms. This model, depicted in Figure 1, was adjusted by a path diagram analysis based on structural equation modelling with the EQS software.

The data showed a moderate adjustment (as shown in Table 3), thus the Lagrange multiplier (LM) test and Wald test values were used to estimate whether constrained or unconstrained parameters could improve the model, provided these were in line with the underlying theory.

The analysis of these parameters established, using Wald test data, that the direct relationship between pessimism and the doctor–patient relationship was not relevant, which is in favour of the indirect effect analysis considering optimism. Similarly, the LM test showed that establishing a direct relationship between pessimism and adverse symptomatology improves the adjustment of the data to the model.

**Figure 2:**
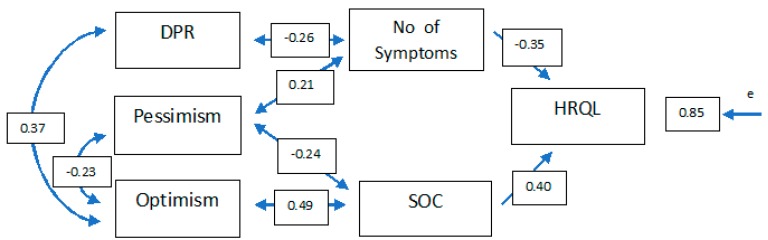
Final model of the relationship between SOC, number of symptoms, and HRQL analysed by contrast. DRP: Doctor–patient relationship; SOC: Sense of coherence; HRQL: Health-related quality of life.

For this reason, a new contrast model was established, which is included in Figure 2 and in which the results show good adjustment indicators (Table 3), making it possible to accept this structure as valid.

As can be seen in the path diagram, the sense of coherence and adverse symptomatology have a very similar burden on health-related quality of life (β = 0.40 and β = −0.35, respectively), showing 28.5% explained variance.

By focusing the analysis on aspects with a higher psychological content, it can be seen that the sense of coherence is more related to optimism (β = 0.49). However, the relationship with pessimism is also significant (β = 0.24), although with less standardised coefficients.

On the other hand, the adverse symptomatology perceived by the patient has a positive correlation with pessimism (β = 0.21) and is negatively related to the doctor–patient relationship (β = 0.26). Finally, there is a significant correlation between optimism and pessimism (β = −0.23) and the doctor–patient relationship (β = 0.37).

## 4. Discussion

Chemotherapeutic treatments are related to a number of important non-haematological toxicities that must be closely monitored by the doctor at each of the patient’s consultation visits prior to administration of the next chemotherapy cycle.

It is important to note the difference in the quality of life of patients with five or fewer toxicities, as compared to those with more than six. However, this significant difference is not as important when it comes to comparing patients with six to ten toxicities and those with more than ten. The reason for this may be due to the psychological change that the emergence of a small number of toxicities causes in a patient who did not present them, as compared to adding certain toxicities in the case of a patient who already suffered them. This can even lead to a decrease in the adherence to the chemotherapy treatment [4].

Generally speaking, the influence of adverse reactions on the quality of life of cancer patients is highly relevant [4]. There are studies [35] that show that the quality of life negatively predisposes to the onset of certain diseases, including breast cancer. Therefore, it can be concluded that a poor quality of life has a negative impact on patient adherence to the treatment process.

Along these lines, there are studies [36,37] that have analysed the quality of life perceived by the patient, as compared to the usual practice. These show that the quality of life is affected by hospital care received during their illness, highlighting the lack of information that health professionals have about patients’ perceptions. Therefore, it would be necessary to raise awareness among healthcare professionals of the importance of adverse reactions related to chemotherapeutic treatments so that they may incorporate a questionnaire as one more section of the daily work with these types of patient [4]. This is considered a prerequisite for informed decision making, although information alone does not ensure that decisions are independent. In shared decision making, options are presented, information is personalized, the concerns and goals of each person are assessed and there is help to ensure decisions are in line with personal levels of risk, values and preferences [38]. In addition, in this way, cancer patients will feel more welcomed by the health system and more participative in everything that affects the evolution of their treatment and disease.

On the other hand, the multivariate relationship model carried out in this study highlights the complex network of relationships between the different constructs analysed in order to explain the impact they have on the quality of life. This study corroborates, together with the results of other research [39], the direct involvement of the sense of coherence on the quality of life, and also adds the number of adverse reactions as a new construct (that had been unproven till now). This implies practically the same impact on the quality of life of these patients but in reverse.

In short, this model shows a network of complex relationships between the different constructs analysed while explaining the adverse symptomatology and quality of life related to health. In this way, the importance of studying the psychological aspects in breast cancer patients has been highlighted, as well as the need to try and alleviate adverse symptoms and foster a good doctor–patient relationship, which would contribute to better levels of quality of life.

Likewise, a greater number of toxicities would be directly involved in a worse doctor–patient relationship and significant levels of pessimism. This relationship would be equally understood in reverse. That is, a worse doctor–patient relationship can directly imply an increase in the patient’s perception of a greater number of toxicities. These data have been corroborated by the results obtained in other studies [4]. The same happens with high levels of pessimism.

In turn, the doctor–patient relationship would be directly related to the number of toxicities (negative relationship) and with optimism (positive relationship). In other words, greater levels of optimism predispose to a better doctor–patient relationship, or expressed backwards, a better doctor–patient relationship leads to greater levels of optimism. In addition, a high quality in the doctor–patient relationship causes the patient to perceive fewer toxicities. According to this model, optimism is indirectly related with the number of toxicities and the involvement made through pessimism or the doctor–patient relationship.

On the other hand, the sense of coherence would be directly affected by both optimism and pessimism, the latter in a negative way. Likewise, the doctor–patient relationship would indirectly affect the sense of coherence through optimism, and the number of toxicities would affect it through pessimism.

Overall, the results of this study support the theory [4] that the coping strategies of patients who, during chemotherapy treatment, suffer a considerable number of toxicities, will end up being affected. This will affect their sense of optimism and even the doctor–patient relationship. All this will lead to a decrease in the quality of life. However, few studies [25] analyse the impact of adverse reactions on the quality of life despite the findings of this study.

As for the limitations of this study, as a single-centre one, there is a possibility that the study population is subjected to some common bias. Likewise, the sample size is adequate although it should be increased for future research so as to clarify certain tendencies expressed through the outcomes obtained in this study. In addition, the questionnaire was carried out randomly during a specific cycle of the six corresponding to the complete treatment, thus the cycle of study may not be adequate for those patients who suffered the most toxicities. On the other hand, no separation has been established between those patients who had received neoadjuvant treatment and those who had received adjuvant treatment, therefore, this could imply a possible risk of bias that must be considered for future research. Similarly, as it is an observational design, causal relationships cannot be established and it is only possible to speak of the correlation between the variables. Therefore, further studies with a larger sample size would be needed to corroborate the results and conclusions of this study. Additionally, the reduced version of the LOT for assessing optimism should be considered as a study limitation. This scale has only 10 items and, although its psychometric properties have been assessed, the reduced number of items may affect the assessment of the concept. It is also worth mentioning that this scale bases its measurement on the perception stated by the patient herself. Likewise, it is recommended to go deeper into the patients’ psychological state by comparing it with their state before the cancer diagnosis so as to also know its evolution throughout the illness.

Overall, health-related life quality data not only aim at helping in decision making on the optimal treatment, but also provide information about the patients’ psychological experience, thus potentially contributing to facilitating the prognosis [40].

Overall, the results of this study show that there is room for improvement in the cancer patient care process (more specifically in people affected by breast cancer). These improvements would imply the inclusion of non-haematological adverse reactions in the oncologist’s clinical decisions through a complete clinical interview with the patient, the incorporation of measures regarding patient data in order to obtain more detailed documentation of the symptoms associated with the treatment, as well as psychological support throughout the chemotherapy treatment process. In this way, there would be a greater sense of acceptance and participation for the patient who needs, more than ever, support from all health professionals (psychologists, doctors, and nurses) related to the field of oncology.

## 5. Conclusions

From this discussion, it can be concluded that in order to improve the quality of life of breast cancer patients it would be necessary to have an impact on the number of adverse reactions involved in the chemotherapeutic treatment, as well as to take into account, during the psychological interventions, the salutogenic approach, with a sense of coherence as a possible starting point. To this end, influencing the quality of a good doctor–patient relationship is proposed, as well as highlighting the importance of psychosocial support for improving optimism to the detriment of pessimism.

## Figures and Tables

**Table 1:** Number of toxicities per patient per cycle.

	Health-Related Quality of Life	
Number of Toxicities	Mean (Valid *n*)	Standard Deviation
5 or less	16.22 (9)	0.83
Between 6 and 10	14.18 (44)	1.54
More than 10	13.56 (25)	2.08

**Table 2:** Sense of coherence, dispositional optimism, and doctor–patient relationship related to the number of toxicities per patient and chemotherapy cycle.

		Sense of Coherence		Optimism		Pessimism		Doctor–patient Relationship	
		M (*n*)	SD	M (*n*)	SD	M (*n*)	SD	M (*n*)	SD
Number of Toxicities	5 or less	69.80 (10)	12.08	13.70 (10)	1.70	7.80 (10)	3.33	44.60 (10)	0.70
	Between 6 and 10	61.37 (43)	12.39	12.79 (43)	1.97	8.07 (43)	3.12	41.32 (44)	4.84
	More than 10	60.43 (21)	11.55	12.54 (24)	2.75	10.33 (24)	2.99	41.63 (24)	5.66

**Table 3:** Adjustment rates of the tested multivariate models.

	Initial Model	Final Model
χ²	7.630	3.301
*P*	0.366	0.856
χ²/df	1.090	0.472
NNFI	0.905	0.959
CI	0.900	0.999
CFI	0.901	0.999
AGFI	0.900	0.955
RMSR	0.069	0.052
RMSEA	0.036	0.001
CI 95%	(0.001–0.152)	(0.001–0.079)
HRQL R^2^	0.284	0.285

χ²: chi-square distribution; *P*: *p* value; NNFI: Non-Normed Fit Index; CI: confidence interval; CFI: Comparative Fit Index; AGFI: adjusted goodness-of-fit index; RMSR: root-mean-square residual; RMSEA: root mean-square error of approximation; HRQL adjusted R^2.^

## Supplementary Materials

The following are available online at https://www.mdpi.com/2077-0383/8/12/2043/s1, Supplementary File 1: Questionnaire.
