# Supplementary material for: Influence of the Sense of Coherence, the Doctor–Patient Relationship, Optimism and Non-Haematological Adverse Reactions on Health-Related Quality of Life in Patients with Breast Cancer
Source: J Clin Med. 2019 Nov 21;8(12):2043. doi: 10.3390/jcm8122043 (PMC6947165; doi:10.3390/jcm8122043)
Supplement: Supplementary file 1 [file jcm-08-02043-s001.pdf]

ANNEX 1. Questionnaire on the oncologist's previous perception of toxicities with the TEC regimen.

Research on adverse symptomatology in the treatment of breast cancer

Dear colleague.
From the Juan Ramón Jiménez Hospital in Huelva, we are carrying out research on the adverse symptomatology associated with the TEC regimen in women with breast cancer.
The questionnaire just includes the chart you can see below, where you are asked to state the frequency with which each of these symptoms is recorded in patients with this type of cancer who are being treated with the TEC regimen.
Thank you in advance for your collaboration. Best regards.

Could you please tell how frequently these symptoms are reported by the women with breast cancer you treat in your clinical practice?

	5% or less	10 %	20 %	30 %	40 %	50 %	60 %	70 %	80 %	90 %	95% or more
**Vomiting**	○	○	○	○	○	○	○	○	○	○	○
**Nausea**	○	○	○	○	○	○	○	○	○	○	○
**Diarrhoea**	○	○	○	○	○	○	○	○	○	○	○
**Constipation**	○	○	○	○	○	○	○	○	○	○	○
**Asthenia**	○	○	○	○	○	○	○	○	○	○	○
**Mucositis**	○	○	○	○	○	○	○	○	○	○	○
**Dysgeusia**	○	○	○	○	○	○	○	○	○	○	○
**Loss of appetite**	○	○	○	○	○	○	○	○	○	○	○
**Hair loss**	○	○	○	○	○	○	○	○	○	○	○
**Skin reaction in hands and/or feet**	○	○	○	○	○	○	○	○	○	○	○
**Nails disorders**	○	○	○	○	○	○	○	○	○	○	○
**Skin allergies**	○	○	○	○	○	○	○	○	○	○	○
**Peripheral neuropathy**	○	○	○	○	○	○	○	○	○	○	○
**Peripheral edema**	○	○	○	○	○	○	○	○	○	○	○
**Headache**	○	○	○	○	○	○	○	○	○	○	○
**Abdominal pain**	○	○	○	○	○	○	○	○	○	○	○
**Joint pain**	○	○	○	○	○	○	○	○	○	○	○
**Muscle pain**	○	○	○	○	○	○	○	○	○	○	○
